# Enhanced Red Color Conversion via Mixing Green and Red Quantum Dots in a Polydimethylsiloxane-Based Color Conversion Layer

**DOI:** 10.3390/mi17070762

**Published:** 2026-06-23

**Authors:** Sang-Uk Byun, Su-Been Lee, Seo-Young Kim, Yu-Lim Seok, Gun Park, Dae-Gyu Moon

**Affiliations:** Department of Electronic Materials, Device, and Equipment Engineering, Soonchunhyang University, Asan-si 31538, Chungcheongnam-do, Republic of Korea

**Keywords:** quantum dot, color conversion layer, mixed QD layer, blue leakage, color conversion efficiency

## Abstract

Quantum dot (QD) color conversion technology has attracted considerable attention in QD-OLED displays because it enables the generation of highly pure red and green emissions from blue OLED excitation with reduced fabrication complexity. Mixed QD color conversion layers consisting of red and green QDs dispersed in a polydimethylsiloxane matrix were fabricated to improve red color conversion efficiency and suppress blue leakage. The color conversion characteristics of the mixed QD layers were investigated by varying the QD contents and layer thicknesses. The color conversion spectra, blue leakage characteristics, color conversion efficiency, and output/input efficiency were systematically analyzed. Compared with red QD-only layers, the mixed QD layers exhibited more effective suppression of blue leakage and stronger red emission even at relatively small layer thicknesses. The red QD-only layer containing 100 mg of red QDs exhibited a color conversion efficiency of 16.7% at a thickness of 38 µm, whereas the mixed QD layer with the same red QD content achieved a higher color conversion efficiency of 19.3% at a thickness of only 5.6 µm. The enhanced color conversion efficiency of the mixed QD layers is likely associated with increased absorption of blue photons and additional excitation of red QDs through photon reabsorption and possible energy transfer processes between the green and red QDs.

## 1. Introduction

Quantum dot (QD)-based color conversion technology has emerged as an attractive approach for realizing high-performance displays owing to narrow emission bandwidth, high color purity, and wavelength tunability of QDs [1,2,3,4,5,6]. In particular, QD color conversion layers integrated with blue organic light-emitting devices (OLEDs) have attracted considerable attention as key components for next-generation QD-OLED displays because they enable efficient generation of red and green emissions while maintaining a simplified device architecture [7,8,9]. Compared with conventional RGB OLED structures, QD color conversion systems provide several advantages including reduced fabrication complexity, improved color gamut, and compatibility with large-area and flexible displays [10,11]. Carbon dots have also attracted increasing interest as a color conversion material owing to their low toxicity, environmental friendliness, facile synthesis, and excellent chemical stability [12,13,14]. However, carbon dots generally exhibit broader emission spectra and their photoluminescence efficiencies are often lower than those of inorganic semiconductor QDs. Therefore, although carbon dots are promising for environmentally friendly optoelectronic applications, inorganic QDs remain advantageous for display applications requiring high color purity and narrow spectral linewidths [12,13,14].

The optical performance of QD color conversion layers is primarily determined by the utilization efficiency of excitation photons emitted from blue OLEDs. Ideally, blue photons should be completely absorbed by QDs and subsequently converted into longer-wavelength emissions. However, in practical color conversion layers, a substantial fraction of blue photons can pass through the QD layer without absorption, resulting in undesirable blue leakage and reduced color conversion efficiency [15,16,17]. Therefore, enhancing the absorption of excitation photons within the QD color conversion layers is crucial for high-performance QD-OLED displays.

Several methods have been proposed for improving the absorption efficiency of QD color conversion layers [18,19,20,21,22,23,24,25,26]. Increasing the thickness or concentration of QDs can improve the absorption of excitation photons [18,19,20,21,22]. Another method for improving the absorption efficiency is to increase the optical path length by utilizing scattering particles such as TiO_2_, BaTiO_3_, and ZnO nanoparticles [23,24,25,26]. However, high QD concentrations can lead to strong inter-dot interactions, resulting in non-radiative recombination and photoluminescence quenching [27,28]. Similarly, excessive thickness may induce self-absorption, scattering losses, and increased material consumption, thereby limiting the overall efficiency of the color conversion processes [29,30].

In this paper, we investigated the optical characteristics of mixed QD color conversion layers consisting of green QDs and red QDs. When the absorption spectrum of larger-sized QDs with a lower bandgap overlaps with the emission spectrum of smaller-sized QDs with a larger bandgap, the excitation energy generated in the larger-bandgap QDs may be transferred to the lower-bandgap QDs through photon reabsorption and/or possible energy transfer processes, resulting in enhanced emission from the lower-bandgap QDs [31,32,33,34]. Therefore, the green QDs can serve as intermediate absorbers that enhance the utilization of blue excitation photons and provide additional excitation pathways for the red QDs. Although such cascade processes have been reported in various QD assemblies, their role in QD-based color conversion layers remains insufficiently understood [35,36]. In particular, the influence of green QDs on the emission characteristics and color conversion efficiency of red QD conversion layers has not been systematically investigated. To address these issues, red and green QDs were incorporated into a polydimethylsiloxane (PDMS) matrix to form mixed QD layers with varying QD compositions and layer thicknesses. The optical characteristics of the mixed QD layers, including emission spectra, blue leakage, color conversion efficiency, and output/input efficiency, were comprehensively evaluated.

## 2. Materials and Methods

Glass substrates coated with ITO were prepared to fabricate blue OLEDs as excitation sources. ITO substrates were patterned using a conventional photolithography process to define the anode electrodes. Acetone and isopropyl alcohol were used to clean the patterned electrodes. The cleaned electrodes were washed with deionized water and subsequently treated with oxygen plasma at 13 W for 3 min. The OLEDs were prepared by sequential evaporation of organic and metal layers under a base pressure below 1 × 10^−6^ Torr. 4,4′-bis(2,2-diphenylethenyl)-1,1′-biphenyl (DPVBi) and N,N′-bis(naphthalen-1-yl)-N,N′-bis(phenyl)benzidine (NPB) were used as the emissive layer and hole transport layer, respectively. The NPB and DPVBi layers were sequentially deposited to thicknesses of 50 nm each. The OLED structure was completed by depositing a LiF electron injection layer and an Al cathode. Finally, the OLEDs were encapsulated in a nitrogen atmosphere.

Red and green oleic acid-capped CdSe/ZnS QDs were used to investigate color conversion characteristics. PDMS and its thermal curing agent were used as the host matrix. The mixed QD layers were prepared by incorporating red and green QDs into a PDMS matrix. QDs were separately dissolved in 1 mL of octane to obtain homogeneous colloidal solutions. The QD solutions were then incorporated into 0.1 g of curing agent and 1 g of PDMS, followed by stirring at 300 rpm for 0.5 h. To investigate the effect of QD composition, red QD content was adjusted to 50 and 100 mg, whereas the amount of green QDs was fixed at 100 mg. For comparison, red QD-only color conversion layers containing 50 or 100 mg of red QDs were also prepared under identical conditions. The prepared mixtures were spin-coated onto glass substrates and cured at 150 °C for 30 min. QD layer thickness was adjusted by varying the repetition number of coating and curing cycles. The fabricated QD layers were placed on the OLEDs, and their color conversion characteristics were evaluated. Transmission electron microscopy (TEM) was used to examine the morphology of QDs (JEOL, JEM-ARM200F, Tokyo, Japan). A surface profilometer was used to measure the thickness of the color conversion layers (Surfcorder ET3000, Kosaka Laboratory, Tokyo, Japan). The electroluminescence (EL) spectra of the OLEDs and the absorption and photoluminescence (PL) spectra of QDs were measured to investigate the optical properties of the QDs. A spectroradiometer (CS1000, Minolta, Tokyo, Japan) was used to measure the emission spectra.

## 3. Results and Discussion

**Figure 1:**
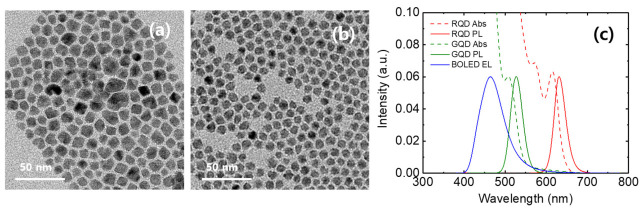
(**a**) TEM image of red QDs, (**b**) TEM image of green QDs, and (**c**) EL spectrum of the blue OLED along with the absorption and PL spectra of red and green QDs.

Figure 1 shows TEM images of the red and green QDs, respectively. TEM images reveal that both red and green QDs possess nearly spherical to slightly faceted polyhedral morphologies with relatively uniform particle sizes. The average particle sizes are 10.3 and 8.0 nm for red and green QDs, respectively. The larger particle size of the red QDs is consistent with the well-known size-dependent optical properties of semiconductor QDs, where larger QDs exhibit longer-wavelength emission due to reduced quantum confinement effects [37,38]. Figure 1c represents the optical properties of the blue OLED and QDs. The red and green QDs exhibit distinct absorption and PL spectra with narrow emission bands in their respective spectral regions. The red and green QDs have emission peaks at 631 and 532 nm, respectively. The full widths at half maximum (FWHMs) of red and green QDs are 33 and 35 nm, respectively. Compared with carbon dots, QDs generally exhibit narrower emission bandwidths and higher PL quantum yields, which are advantageous for achieving high color purity and efficient color conversion [12,13,14]. The blue OLED has a maximum intensity at 465 nm and an FWHM of 69 nm. The absorption bands of both red and green QDs show substantial overlap with the EL spectrum of the OLED, allowing efficient excitation of the QDs and subsequent color conversion. Furthermore, a substantial spectral overlap is observed between the absorption spectrum of the red QDs and the emission spectrum of the green QDs. This overlap suggests that photons emitted from the green QDs can be reabsorbed by neighboring red QDs and may also facilitate energy transfer processes within the mixed QD color conversion layer [31,32,33,34,35,36]. Such cascade excitation mechanisms are expected to enhance the color conversion characteristics of the mixed QD system.

**Figure 2:**
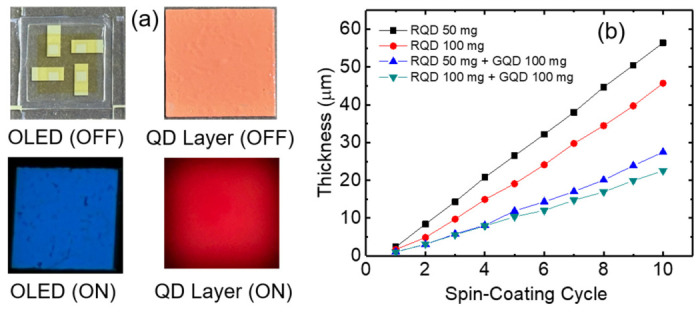
(**a**) Photographs of the OLED and QD conversion layer samples in the on and off states and (**b**) dependence of layer thickness on coating cycle for different QD contents.

Figure 2 shows the OLED and QD color conversion layer samples in on and off states. The emission photographs of the OLED and color conversion layer were taken in a light-tight dark box. The OLED exhibits a uniform blue emission over the entire active area. The QD color conversion layer appears orange-red in the off state and emits bright red light under blue OLED excitation. The red emission exhibits good spatial uniformity over the entire active area without noticeable dark spots or intensity fluctuations. No obvious emission non-uniformity associated with QD aggregation is observed, suggesting that the mixed QDs are homogeneously dispersed within the PDMS matrix. In addition, a slight spreading of the emitted light is observed near the edges of the sample, which is attributed to light scattering by the QDs dispersed in the PDMS matrix [39]. Figure 2b shows the variation in QD layer thickness with the number of spin-coating cycles for different QD compositions. For all samples, the layer thickness increases approximately linearly with increasing number of coating cycles, suggesting that the previously cured layer remains stable during subsequent spin-coating and curing steps. However, despite the higher solid loading, the thickness decreases with increasing QD content. This behavior is likely associated with the oleic acid ligands attached to the QD surfaces. Increasing the QD content simultaneously increases the amount of oleic acid in the PDMS matrix, which can act as a plasticizer and reduce the viscosity of the PDMS-QD solution. The lower viscosity facilitates radial flow during spin-coating, leading to the formation of thinner layers. Similar viscosity-reduction behavior induced by oleic acid has been reported previously [39,40,41].

**Figure 3:**
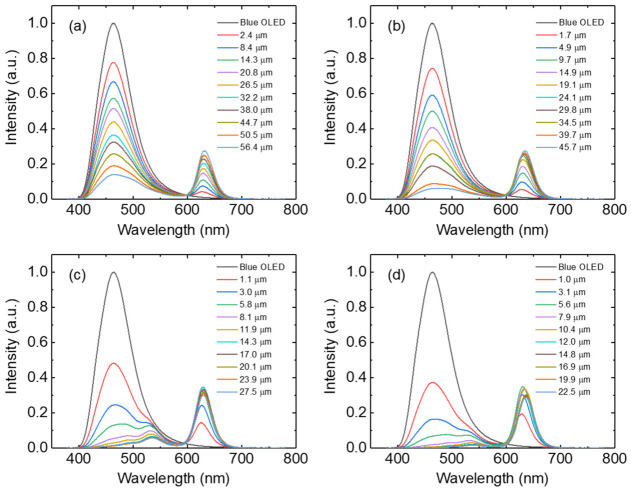
Emission spectra of the color conversion layers containing (**a**) 50 mg of red QDs, (**b**) 100 mg of red QDs, (**c**) 50 mg of red QDs and 100 mg of green QDs, and (**d**) 100 mg of red QDs and 100 mg of green QDs.

Figure 3a presents the emission spectra for the red QD-only layer prepared by incorporating 50 mg of red QDs in 1 g of PDMS. As the layer thickness increases, blue light absorption by the QDs increases, resulting in an increase in red emission from the QDs and a decrease in the transmitted blue emission. However, a significant amount of blue light emission remains even at a thickness of 56.4 µm. When the red QD content increases to 100 mg, the residual blue emission, i.e., blue leakage, is reduced even at thinner thicknesses, while the red emission increases.

The emission spectra of the mixed QD layer fabricated by dispersing 50 mg of red QDs and 100 mg of green QDs in 1 g of PDMS are shown in Figure 3c. Compared with the red QD-only layer, the mixed QD layer shows enhanced red emission and significantly reduced blue leakage even at smaller thicknesses. Furthermore, increasing the red QD content to 100 mg further suppresses blue leakage, while substantially enhancing the red emission even at relatively small layer thicknesses. These results indicate that the green QDs increase the overall absorption of blue photons and provide an additional excitation pathway for red QDs through photon reabsorption and possible Förster resonance energy transfer (FRET) processes [31,32,33,34,35,36]. Consequently, the mixed QD structure enables more efficient utilization of the blue OLED emission. Regarding the energy transfer mechanism, FRET is considered more plausible than Dexter transfer in the mixed QD system [31,32]. As shown in Figure 1c, the green QD emission exhibits a significant overlap with the absorption spectrum of the red QDs. In addition, since the QDs with organic ligands are dispersed in a PDMS matrix, the interparticle distances are expected to be sufficiently large to suppress Dexter transfer.

**Figure 4:**
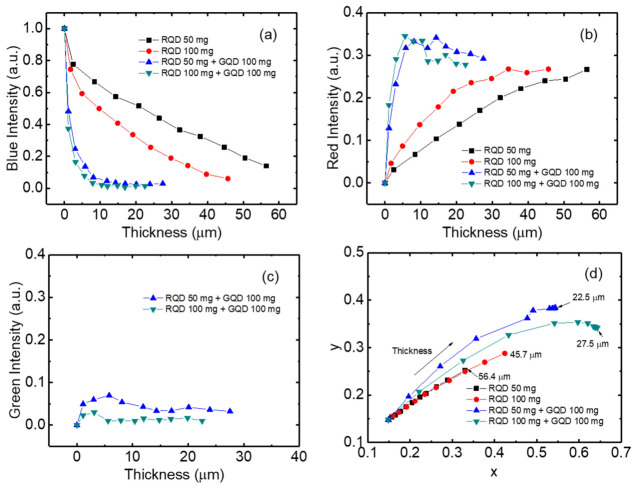
(**a**) Blue, (**b**) red, and (**c**) green emission intensities, and (**d**) CIE color coordinates of red QD-only and mixed QD color conversion layers.

Figure 4a shows the normalized blue emission intensity for the color conversion layers. For all samples, the blue emission decreases with increasing layer thickness due to the increased optical path length of blue photons. For example, the red QD-only color conversion layer containing 50 mg of red QDs exhibits a blue intensity of 0.78 at a thickness of 2.4 µm, which decreases to 0.37 as the thickness increases to 32.2 µm. When the red QD content increases to 100 mg, the absorption of blue photons by the QDs becomes stronger, resulting in a more rapid decrease in blue intensity with increasing thickness. The mixed QD layers exhibit a much more rapid suppression of blue emission compared with the red QD-only layers, indicating that the incorporation of green QDs significantly enhances the absorption of blue light. Figure 4b shows the variation in red emission peak intensity. In the red QD-only layers, the red emission gradually increases with increasing layer thickness because of the enhanced absorption of blue photons. When the red QD content increases, the red intensity becomes stronger because a larger number of QDs absorb blue photons. The mixed QD layers exhibit much stronger red emission than the red QD-only layers at thinner thicknesses. These results suggest that the mixed QD structure improves the utilization of blue photons and promotes additional excitation of red QDs through photon reabsorption and possible FRET processes between the green and red QDs.

Figure 4c shows the peak intensity of green emission in the mixed QD layers as a function of layer thickness. As green QDs are incorporated into the mixed layer, blue photons are absorbed by both red and green QDs, resulting in green emission in addition to red emission. However, despite the addition of 100 mg of green QDs, the emission intensity of the green QDs remains much lower than that of the red QDs because of photon reabsorption and possible FRET to the red QDs from the green QDs. As the red QD content increases from 50 to 100 mg, the photon reabsorption or FRET from the green to the red QDs becomes more pronounced, resulting in a lower green emission intensity. Figure 4d shows the corresponding CIE color coordinates. As the layer thickness increases, the color coordinates gradually shift away from the blue region toward the red region, reflecting the suppression of blue leakage and the enhanced color conversion by the QDs. As the red QD content increases, the QDs absorb a larger number of blue photons and convert them into red emission more effectively, shifting the color coordinates toward the red region. However, the color coordinates are still located relatively far from the red region at (0.42, 0.29) even at a thickness of 45.7 µm. Compared with the red QD-only layers, the mixed QD layers exhibit a more pronounced shift toward the red color region with reduced blue contribution. For example, the mixed QD layer containing 50 mg of red QDs reaches (0.53, 0.38) at a thickness of 27.5 µm. When the red QD content increases to 100 mg, the color coordinates further shift toward the red region to (0.64, 0.34) at a thickness of 22.5 µm.

**Figure 5:**
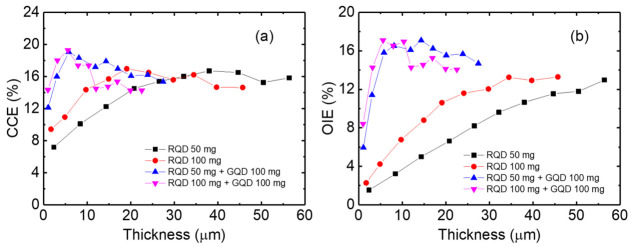
(**a**) Color conversion efficiency; (**b**) output/input efficiency for the QD color conversion layers.

Figure 5a shows the variation in color conversion efficiency with layer thickness for the different QD compositions. The color conversion efficiency represents the fraction of absorbed blue light that is converted into red emission [16,39]. As the layer thickness increases, the color conversion efficiency increases, followed by saturation or a slight decrease. This result is attributed to the insufficient absorption of blue photons in thin layers, whereas thicker layers enhance blue light absorption by the QDs, thereby improving color conversion. However, beyond a certain thickness, most of the blue photons are already absorbed, reducing the effect of further thickness increase. At the same time, self-absorption and scattering losses become more significant, resulting in efficiency saturation or a slight decrease. The red QD-only layer containing 50 mg of red QDs achieves a 16.7% conversion efficiency at 38 µm. When the red QD content increases to 100 mg, the absorption of blue photons by the QDs increases, and the efficiency increases to 17.0% at 19.1 µm. The mixed QD layers show a much steeper increase in color conversion efficiency with thickness and exhibit overall higher efficiency than the red QD-only layers. The mixed QD layer containing 50 mg of red QDs and 100 mg of green QDs achieves a conversion efficiency of 19.0% at 5.8 µm, while the mixed QD layer containing 100 mg of red QDs and 100 mg of green QDs obtains an efficiency of 19.3% at 5.6 µm. These values are substantially higher than the red QD-only layers with the same red QD content. Figure 5b shows the variation in the output/input efficiency with layer thickness for the different QD compositions. The output/input efficiency represents the ratio of the converted red light to the incident blue light [16,39]. In the red QD-only layers, the efficiency gradually increases with increasing layer thickness. In contrast, the mixed QD layers exhibit higher efficiency even at relatively small thicknesses, and the efficiency slightly decreases with further increases in thickness. Since the output/input efficiency is calculated with respect to the total incident blue light rather than the absorbed blue light, its values are inherently lower than those of the color conversion efficiency [16,39]. The output/input efficiencies are 13.0% and 13.3% at 56.4 and 45.7 µm, respectively, in the red QD-only layers containing 50 and 100 mg of red QDs. In contrast, the mixed QD layers containing 50 and 100 mg of red QDs exhibit higher output/input efficiencies of 17.1% at 14.3 and 5.6 µm, respectively. Notably, the mixed QD layers achieved higher conversion and output/input efficiencies at significantly smaller thicknesses than the red QD-only layers, demonstrating the effectiveness of the mixed-QD strategy for realizing thin and efficient color conversion layers. For comparison, recent InGaN red LEDs have demonstrated peak external quantum efficiencies exceeding 10% [42]. Although the efficiency metrics of color conversion layers cannot be directly compared with those of self-emissive InGaN-based LEDs because of their fundamentally different operating principles, the mixed QD color conversion approach provides an alternative strategy for achieving efficient red emission with simplified device architectures.

## 4. Conclusions

The color conversion characteristics of the mixed QD layers containing the red and green QDs were investigated. The amount of red QDs dispersed in the PDMS matrix was set to 50 and 100 mg, while the content of green QD was fixed at 100 mg. The mixed QD layers substantially suppressed the blue emission and enhanced the red emission even at relatively small layer thicknesses. The incorporation of green QDs improved the harvesting of blue excitation photons and promoted the additional excitation of the red QDs through photon reabsorption and possible energy transfer between the green and red QDs. Consequently, the mixed QD layers exhibited a more pronounced shift in the CIE color coordinates toward the red region as the layer thickness increased. In addition, the mixed QD layers showed higher efficiencies than the red QD-only layers at smaller thicknesses due to enhanced photon utilization within the mixed QD structure. The red QD-only layer containing 100 mg of red QDs exhibited a 17.0% color conversion efficiency at 19.1 µm and a 13.3% output/input efficiency at 45.7 µm. In contrast, the mixed QD layer containing the same amount of red QDs achieved corresponding efficiencies of 19.3% and 17.1%, respectively, both at a thickness of only 5.6 µm. These results demonstrate that the mixed green and red QD structure is an effective strategy for suppressing blue leakage and enhancing red color conversion performance in QD-OLED displays.

## Data Availability

Data are contained within the article.

## References

[1] Alivisatos A.P. (1996). Semiconductor clusters, nanocrystals, and quantum dots. Science.

[2] Reiss P., Rrotière M., Li L. (2009). Core/shell semiconductor nanocrystals. Small.

[3] Shirasaki Y., Supran G.J., Bawendi M.G., Bulović V. (2013). Emergence of colloidal quantum-dot light-emitting technologies. Nat. Photonics.

[4] Arquer F.P.G., Talapin D.V., Klimov V.I., Arakawa Y., Bayer M., Sargent E.H. (2021). Semiconductor quantum dots: Technological progress and future challenges. Science.

[5] Jang E., Jang H. (2023). Review: Quantum dot light-emitting diodes. Chem. Rev..

[6] Kim J., Roh J., Park M., Lee C. (2024). Recent advances and challenges of colloidal quantum dot light-emitting diodes for display applications. Adv. Mater..

[7] Huang Y.M., Singh K.J., Liu A.C., Lin C.C., Chen Z., Wang K., Lin Y., Liu Z., Wu T., Kuo H.C. (2020). Advances in quantum-dot-based displays. Nanomaterials.

[8] Li G., Tseng M.C., Chen Y., Yeung F.S., He H., Cheng Y., Cai J., Chen E., Kwok H.S. (2024). Color-conversion displays: Current status and future outlook. Light Sci. Appl..

[9] Chen J., Zhao Q., Yu B., Lemmer U. (2024). A review on quantum dot-based color conversion layers for mini/micro-LED displays: Packaging, light management, and pixelation. Adv. Opt. Mater..

[10] Kim D.Y., Han Y.J., Choi J., Sakong C., Ju B.K., Cho K.H. (2020). Inkjet printed quantum dot film formed by controlling surface wettability for blue-to-green color conversion. Org. Electron..

[11] Hu Z., Yin Y., Ali M.U., Peng W., Zhang S., Li D., Zou T., Li Y., Jiao S., Chen S.J. (2020). Inkjet printed uniform quantum dots as color conversion layers for full-color OLED displays. Nanoscale.

[12] Trapani D., Macaluso R., Crupi I., Mosca M. (2022). Color conversion light-emitting diodes based on carbon dots: A review. Materials.

[13] Li C.X., Yu C., Wang C.F., Chen S. (2013). Facile plasma-induced fabrication of fluorescent carbon dots toward high-performance white LEDs. J. Mater. Sci..

[14] Da X., Han Z., Yang Z., Zhang D., Hong R., Tao C., Lin H., Huang Y. (2022). Preparation of multicolor carbon dots with high fluorescent quantum yield and application in white LED. Chem. Phys. Lett..

[15] Yeo H.J., Yoon S.Y., Jo D.Y., Kim H.M., Kwak J., Kim S.P., Kim M.J., Yang H. (2022). Effective blue light-absorbing AuAg nanoparticles in InP quantum dots-based color conversion. Materials.

[16] Park H., Hahm D., Cho H., Shin J.W., Kang C.M., Ahn D.H., Joo C.W., Kwon B.H., Kim K., Kim J.Y. (2023). Efficient quantum dot color conversion layer with mixed spherical/rod-shaped scattering particles. ACS Appl. Opt. Mater..

[17] Lin Y., Huang W., Zhanghu M., Liu Z. (2023). Ultra-thick inkjet-printed quantum dots layer for full-color micro-LED displays. Opt. Express.

[18] Hens Z., Moreels I. (2012). Light absorption by colloidal semiconductor quantum dots. J. Mater. Chem..

[19] Xu S., Yang T., Lin J., Shen Q., Li J., Ye Y., Wang L., Zhou X., Chen E., Ye Y. (2021). Precise theoretical model for quantum-dot color conversion. Otp. Express.

[20] Wang Y., Luo Y., Kong X., Wu T., Lin Y., Chen Z., Wang S. (2025). Patterning technologies of quantum dots for color-conversion micro-LED display applications. Nanoscale.

[21] Zhou S., Li Y., Gong Z. (2023). Wafer-scale patterning of high-resolution quantum dot films with a thickness over 10 µm for improved color conversion. Nanoscale.

[22] Kim H.M., Ryu M., Cha J., Kim H.S., Jeong T., Jang J. (2019). Ten micrometer pixel, quantum dots color conversion layer for high resolution and full color active matrix micro-LED display. J. Soc. Inf. Disp..

[23] Tang Y., Li Z., Li Z.T., Li J.S., Yu S.D., Rao L.S. (2018). Enhancement of luminous efficiency and uniformity of CCT for quantum dot-converted LEDs by incorporating with ZnO nanoparticles. IEEE Trans. Electron Devices.

[24] Lin S., Tan G., Yu J., Chen E., Weng Y., Zhou X., Xu S., Ye Y., Yan Q.F., Guo T. (2019). Multi-primary-color quantum-dot down-converting films for display applications. Opt. Express.

[25] Yang H., Zhou M., Tang H., Sun M., Liu P., Liu Y., Chen L., Li D., Wu D., Hao J. (2020). Enhanced light emission of quantum dot films by scattering of poly(zinc methacrylate) coating CdZnSeS/ZnS quantum dots and high refractive index BaTiO_3_ nanoparticles. RSC Adv..

[26] Hyun B.R., Sher C.W., Chang Y.W., Lin Y., Liu Z., Kuo H.C. (2021). Dual role of quantum dots as color conversion layer and suppression of input light for full-color micro-LED displays. J. Phys. Chem. Lett..

[27] Tai K., Lu W., Umezu I., Sugimura A. (2010). Inter-dot distance dependence of photoluminescence properties in CdSe quantum dot systems. Appl. Phys. Express.

[28] Vatassery R., Hinke J.A., Hue A.S.R., Mann K.R., Blank D.A., Gladfelter W.L. (2013). Excited-state quenching mechanism of a terthiophene acid dye bound to monodisperse CdS nanocrystals: Electron transfer versus concentration quenching. J. Phys. Chem. C.

[29] Zeng Y., Qian W., Long R., Zhang X., Ma F., Luo J., Lian L., Zhang D., Zhang J. (2025). Full-color micro-LED displays based on quantum dot color converters. Nano Res..

[30] Lin P., Ji X., Wang X., Yin L., Zhang J. (2023). Quantum dot color conversion characteristics and performance improvement based on micro-LED. IEEE Trans. Electron Devices.

[31] Kagan C.R., Murray C.B., Nirmal M., Bawendi M.G. (1996). Electronic energy transfer in CdSe quantum dot solids. Phys. Rev. Lett..

[32] Chou K.F., Dennis A.M. (2015). Förster resonance energy transfer between quantum dot donors and quantum dot acceptors. Sensors.

[33] Chen C.J., Lin C.C., Lien J.Y., Wang S.L., Chiang R.K. (2015). Preparation of quantum dot/polymer light conversion films with alleviated Förster resonance energy transfer redshift. J. Mater. Chem. C.

[34] Yuan Y., Chen Z., Guo Z., Wang H.Q., Geng C., Xu S. (2025). Study of energy transfer dynamic in mixed quantum dots for on-chip white light-emitting diodes. ACS Photonics.

[35] Rowland C.E., Fedin I., Zhang H., Gray S.K., Govorov A.O., Talapin D.V., Schaller R.D. (2015). Picosecond energy transfer and multiexciton transfer outpaces Auger recombination in binary CdSe nanoplatelet solids. Nat. Mater..

[36] Li B., Li Q., Zhu Z., Li K., Lin F., Shi Y., Wei Z. (2024). Enhancing Förster resonance energy-transfer rates in perovskite quantum dots via controlling donor-acceptor ratios. J. Phys. Chem. C.

[37] Brus L.E. (1984). Electron-electron and electron-hole interactions in small semiconductor crystallites: The size dependence of the lowest excited electronic state. J. Chem. Phys..

[38] Hu R., He F., Hou R., Wu Z., Zhang X., Shen H. (2024). The narrow synthetic window for highly homogeneous InP quantum dots toward narrow red emission. Inorg. Chem..

[39] Byun S.W., Lee S.B., Kim S.Y., Seok Y.L., Park G., Moon D.G. (2026). Polydimethylsiloxane-based quantum dot color conversion layers for QD-OLED applications. Micromachines.

[40] Sander M.M., Nicolau A., Guzatto R., Samios D. (2012). Plasticiser effect of oleic acid polyester on polyethylene and polypropylene. Polym. Test..

[41] Liu L., He S., Tang L., Yang S., Ma T., Su X. (2021). Application of CO2-switchable oleic-acid-based surfactant for reducing viscosity of heavy oil. Molecules.

[42] Zheng X., Zhao G., Dai Y., Fu Y., Zhou M., Huang T., Tan S.T., Sharma V.K., Lu Y., Wu T. (2025). V-pit-induced electric field distribution enabling efficient hole injection in InGaN-based red light-emitting diodes grown on silicon. PhotoniX.

